# Exploring emotional expression in invertebrates: mechanisms, functions and phylogeny

**DOI:** 10.1098/rstb.2025.0146

**Published:** 2026-09-24

**Authors:** Luigi Baciadonna, Catherine Macri, David Baracchi, Martin Giurfa

**Affiliations:** ^1^ CNRS, Inserm, Institut de Biologie Paris-Seine, IBPS, Sorbonne Université, Paris, France; ^2^ CNRS, Inserm, Centre de Neuroscience Neuro-SU, Sorbonne Université, Paris, France; ^3^ Department of Life Sciences and Systems Biology, University of Turin, Turin, Italy; ^4^ School of Arts and Cultures, Newcastle University, Newcastle upon Tyne, UK; ^5^ Dipartimento di Biologia, Università degli Studi di Firenze Scuola di Scienze Matematiche Fisiche e Naturali, Sesto Fiorentino, Firenze, Italy

**Keywords:** affective neuroscience, biogenic amines, cognitive bias, emotional states, insects

## Abstract

The idea that invertebrates may exhibit basic forms of emotion, and possibly sentience, is gaining traction as evidence of their advanced cognitive abilities continues to mount. While high-level cognition does not imply emotional states, invertebrates offer a unique window into whether such states can arise in small-brained animals. With their relatively simple yet highly structured nervous systems, they serve as tractable models for dissecting the mechanisms underlying emotional expression. To probe these processes, researchers have developed behavioural paradigms exposing different species to contexts likely to elicit emotional responses. From a phylogenetic perspective, studying emotional expression in invertebrates can reveal whether basic emotional systems are conserved across species. This review synthesizes current knowledge of non-verbal emotional expression in social insects, focusing on mechanisms and functional roles. We apply a multi-component approach to examine key building blocks shared across emotions and taxa, offering insights into their evolutionary origins. We also identify critical gaps in knowledge and propose directions for future research. By examining the proximate and ultimate causes of emotion, this framework enhances our understanding of the biological basis and evolutionary significance of emotions, promoting interdisciplinary research that bridges neuroscience, ethology and evolutionary biology.

This article is part of the theme issue ‘Mechanisms, development, phylogeny and functions of emotional expressions’.

## 1. Introduction

For a long time, the study of invertebrate behaviour has been restricted to stereotyped responses, based on the assumption that their miniature brain rendered them capable only of executing rigid, repetitive routines [1]. In recent years, however, this perspective has been challenged, and invertebrates have emerged as powerful models to investigate the mechanisms underlying complex behaviours. Invertebrates have been used to characterize the neural circuits of sleep, courtship, social foraging and simple forms of associative learning and memory [2–7]. Moreover, accumulating evidence shows that invertebrates are capable of experience-dependent plasticity and cognitive abilities that go beyond simple associative learning and that were once thought to be exclusive to vertebrates. These include concept learning [8,9], numerical cognition [10–12], stimulus categorization [13,14], behavioural flexibility [15,16] and even rudimentary forms of cultural transmission [17,18].

Cognitive sophistication does not, by itself, imply emotional experience; yet the growing recognition of invertebrate intelligence has prompted the compelling debate as to whether invertebrates experience basic emotional states. In this review, we will appraise the evidence for emotional experiences in invertebrates. However, before the evidence can be critically assessed, it is essential to first clarify what is meant by ‘emotion’ and explain why studying emotions in non-human animals, including invertebrates, is scientifically meaningful. We argue that invertebrates offer not only a window into the building blocks of cognition and behaviour, but also into the fundamental mechanisms underlying emotional experience.

## 2. Defining emotion: a functional approach

Emotions are among the most vivid and immediate aspects of our mental lives, yet they remain elusive to define in a single, comprehensive way [19–21]. Emotions are transient central states that induce coordinated changes across subjective experience, cognition, behaviour and physiology, and are triggered by the appraisal of environmental stimuli that are salient to the individual's goals, needs and well-being [19,20,22–24]. Motivation, closely linked to emotion, refers to the internal processes that initiate, direct and sustain goal-directed behaviour, providing the drive behind emotion-driven actions [25]. By contrast, mood refers to a more enduring and diffuse affective condition, typically lower in intensity and lacking a specific eliciting stimulus, which reflects the cumulative integration of recent emotional episodes and represents an individual's overall position along the dimensions of valence and arousal [19,26]. Emotions and mood are often considered components of the broader construct of affect [19,26–28]. In general, emotions are complex, multi-faceted phenomena that can be examined from multiple perspectives. Consider, for instance, encountering a grizzly bear while hiking. The resulting human emotion could be widely defined as ‘fear’ and characterized by multiple reactions, including a cognitive evaluation (‘this is dangerous!’), physiological changes (e.g. increased heart rate), a phenomenological experience (e.g. a feeling of terror), expressive changes (e.g. widened eyes, tense facial expression), behavioural tendencies (e.g. fleeing) and cognitive shifts (e.g. heightened attention).

From a biological and evolutionary standpoint, emotions are widely understood as functional neural mechanisms that enhance adaptive responses to environmental challenges, providing flexibility beyond simple reflexes while remaining more efficient than slow, deliberative behaviour [23,29,30]. Because emotions span such diverse experiential and functional domains, different theoretical approaches have often focused on specific components in isolation, rather than offering an integrated account [31,32]. For example, the *feeling tradition* emphasizes the conscious experience of emotions; the *evaluative tradition* focuses on the cognitive appraisals that underlie them and the *motivation tradition* highlights action-oriented functions [31]. However, despite their differences, all three traditions have tried to provide answers to common core questions in the study of emotions: how are emotions distinguished from one another and from non-emotional states (differentiation)? How do emotions initiate and impact behavioural decisions (motivation)? What is the object-directed nature of emotions (intentionality)? And what do emotions feel like (phenomenology)?

Over time, definitions of emotion have been enriched by research into physiological markers, somatic responses and cognitive processes [19,20,22]. This broader, multi-component perspective enables the integration of these various viewpoints within a single conceptual framework. It also opens the door to comparative approaches, allowing scientists to investigate such processes in phylogenetically distant species, including invertebrates, where emotional expressions may not be homologous to those in humans but can be functionally analogous [24]. Research across species is often guided by the idea that they may share basic functional building blocks that are conserved across emotions and taxa [24,29,30]. These include persistence (emotional states that outlast the eliciting stimulus), scalability (responses varying in intensity with the stimulus strength), valence (positive or negative affective tone), generalization (transfer of responses to similar stimuli), global coordination (engage the whole organism), automaticity (rapid, involuntary modulation of behaviour) and social communication (signals that influence or inform others). Focusing on these building blocks helps distinguish emotions from simple reflexes and emphasizes their adaptive, functional role in guiding behaviour [30]. It also shifts the debate away from whether or not a species ‘has’ emotions and towards understanding the mechanisms and biological significance of affective processes [24].

The subjective experience of emotion, the qualitative ‘feeling’, is central but difficult to study. We therefore, for the time being, set consciousness aside (see a recent contribution on this aspect [33]) and focus on tractable components such as physiological responses, observable behaviours and neural circuits [29,30,34]. This strategy mirrors approaches in vision and in memory research, where mechanisms were analysed before addressing subjective elements. Such an approach is necessary because conscious experience, often referred to as the ‘hard problem’, remains difficult to quantify [35,36]. Moreover, emotions can be triggered by subliminal stimuli, showing that consciousness and emotion do not always coincide [37,38]. By separating emotions from feelings, we do not rule out the possibility that a wide range of animals, including invertebrates, possess some basic forms of subjective experience [32,39,40].

In this review, we focus on the emerging body of research investigating emotional expression in invertebrates through a multi-componential lens. Building on recent efforts to operationalize emotions, we consider studies that assess behavioural, physiological and neurophysiological components. We do not address research on pain perception, which has been extensively reviewed elsewhere [41–43]. Through the lens of invertebrates, we begin by reviewing strengths and limitations of behavioural approaches, cognitive perspective and finally neurophysiological methods, before discussing theoretical and practical implications for future research.

## 3. Behavioural approach

Behaviour provides a direct and accessible window into the emotional lives of animals. In vertebrates, a wide range of behavioural indicators associated with emotions has been described, including whole-body behaviours such as approach–avoidance, freezing and play [19,20,26,28,44,45], as well as more specific measures such as postures, facial expressions, vocalizations and lateralized responses [46–49]. These studies demonstrate not only the richness of behavioural expressions of emotion but also the interpretative challenges they pose. For instance, a given behaviour may arise in multiple emotional contexts, and high-arousal and negative valence states are generally easier to detect than low-arousal and positive valence states [19,26]. A further caveat is that it is often difficult to distinguish behaviours that reflect emotions from those driven by non-emotional processes [19,26]. Nevertheless, the behavioural indicators developed in vertebrates provide a useful starting point for studying invertebrates, but interpretations must be made with caution, taking into account their specific sensory and motor capabilities [50]. The following section outlines key behavioural measures that have been used to evaluate emotional states in invertebrates.

**Figure 1.**
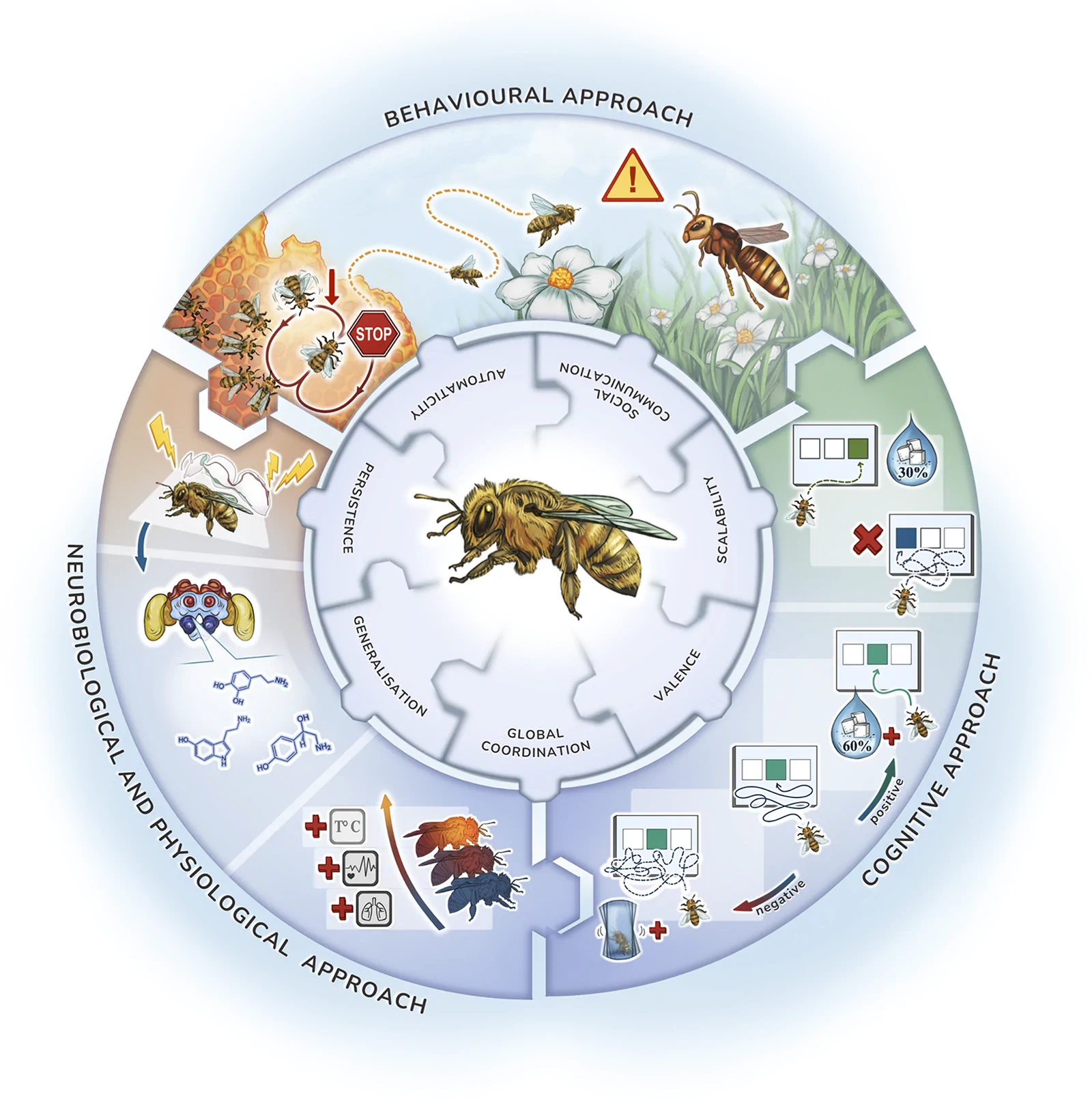
Representation of the three main approaches used to study the expression of emotions. The behavioural approach focuses on observable indicators such as approach or avoidance, posture, facial expressions and sound production. For example, a foraging bee encountering a hornet near a flower patch will rapidly flee and, upon returning to the hive, emit a stop signal that inhibits recruitment dances to that location. The cognitive approach assesses how an emotional stimulus impacts decision-making. In a typical experiment, a bee is trained to associate the colour green on the right with a sugar reward, while blue on the left is unrewarded. When subsequently presented with an intermediate colour, a bee that receives an unexpected high-value sugar reward before making a decision approaches the ambiguous stimulus quickly. By contrast, bees exposed to a stressor prior to the decision are slower to approach the cue than control bees. The neurobiological and physiological approach examines changes induced by emotional triggers in parameters such as body temperature, heart rate or respiration, as well as the modulation of biogenic amine levels in the brain and neurohormones in the haemolymph or periphery. Together, these approaches allow the identification of seven proposed ‘building blocks’ (inner circle) that characterize and differentiate emotions from reflexive or hard-wired responses [23,28].

Animals, including humans, face threats that may cause harm or death, making it essential to learn to avoid cues predicting such threats. Such associations can be acquired via classical conditioning, where a neutral stimulus predicts an aversive event. Early invertebrate studies by the Kandel lab on sea slugs (*Aplysia californica*) showed that aversive conditioning enhanced defensive behaviours [51]. The observed responses included head and siphon withdrawal, inking, escape locomotion and reduced feeding, a pattern which resembles behaviours associated with conditioned fear in mammals [52,53]. Crucially, however, these defensive responses alone, which can be found in different forms in many invertebrates subjected to aversive conditioning (for an extensive review of fear learning in invertebrates, including *A. californica*, *Drosophila melanogaster* and *Caenorhabditis elegans*, see [54]), cannot be taken as evidence of emotional states [53,55,56]. Only when interpreted within a functional, multi-component framework, do such behaviours gain significance as potential affective indicators [24,30]. This framework marks a clear paradigm shift, from a narrow view that equates behaviour with emotion, to an integrative model in which defensive reactions are understood as part of a broader emotional architecture. Gibson *et al.* [57] applied this approach to investigate fear-like responses in fruit flies, using an overhead shadow as an innately aversive stimulus. By quantifying multiple behavioural measures, including locomotor velocity, hopping and freezing, they demonstrated that responses were both scaled and persistent. Furthermore, flies also dispersed from food sources after repeated shadow exposure, indicating generalization across contexts and negative valence. This study highlights the need to assess multiple behavioural criteria (i.e. automaticity, persistence, generalization, global coordination, valence, scalability and social communication) to distinguish emotional states from simple reflexive or environmentally induced responses. Similar principles apply to honey bees (*Apis mellifera*), in which fear-related behaviours can be investigated in a predator context [58]. Hornets like *Vespa velutina* drive colony decline through intense predation and by discouraging workers from foraging (figure 1) [59]. In group-level fear assays, prolonged exposure led bees to cluster away from the predator, with few crossing food areas [60,61]. This adaptive behaviour potentially indicates an internal state modulating both individual and collective decisions, even before direct harm occurs.

Anxiety is a negative emotional state closely related to fear at both behavioural and neurobiological levels, but generally regarded as more complex, as it arises without an immediate threat [62–65]. In vertebrates, anxiety is typically assessed through behavioural responses to potentially aversive contexts, including open spaces or predator cues [66–69]. A widely used method is the elevated plus maze, where the time spent in open versus closed arms indicates anxiety, reflecting rodents’ preference for dark, enclosed areas. Similar paradigms have been applied to crayfish (*Procambarus clarkii*), which also have a preference for darker areas [70]. Crayfish previously exposed to electric shocks or social harassment showed reduced exploration and increased avoidance of open arms, with altered latency, duration and frequency of visits [71]. This avoidance of open areas, along with neurophysiological changes discussed later, reflects the anticipation of potential danger rather than a reaction to an immediate threat, thus representing anxiety rather than fear.

Whereas fear and anxiety reflect responses to immediate or potential threats, repeated exposure to uncontrollable, inescapable stressors can induce a distinct state in which animals cease to avoid aversive stimuli, a phenomenon known as learned helplessness [72,73]. When control is lost, behaviour shifts from active defence or avoidance to a ‘give-up’ state, marked by reduced coping and heightened passivity [74,75]. Early work in fruit flies demonstrated that inescapable stress induces escape deficits in subsequent learning tasks [76]. For instance, flies exposed to inescapable mechanical shaking increased escape latencies in Y-mazes. Yang *et al.* [77] confirmed these observations in a heat-box paradigm—while a master fly could shut down heat stress by walking, a yoked fly could not. Yoked flies moved more slowly, paused more often and escaped less effectively. Batsching *et al.* [78] used electric shocks as the stressor, replicating and extending previous findings. Such effects of uncontrollability are context-specific, affecting persistent locomotion but not courtship or open-field walking. However, caution is warranted before interpreting such behavioural changes as a central emotional state rather than context-dependent adjustments. To fully capture the complexity of fruit fly responses, additional behavioural and neurophysiological measures should be integrated within a multi-component framework, providing a more complete understanding of emotion-like phenomena (see box 1 for details). To date, the term ‘learned thanatosis’ may be preferable to ‘learned helplessness’, as it captures the behavioural phenomenon without implying excessive cognitive complexity or anthropocentric interpretations [75].

Research on animal emotions has largely focused on negative rather than positive emotional states [34]. This trend, well-documented in vertebrates, also characterized the emerging study of invertebrate emotions, where most work addressed fear, anxiety or aversion. Such states are easy to induce and yield robust and measurable responses. Yet, this imbalance may also reflect two underlying biases. Evolutionarily, emotions likely evolved for survival, favouring responses to danger over reward [79–82]. From a psychological perspective, human research has similarly concentrated on negative states because of their disruptive impact on daily life; however, positive states can also be studied. Solvi *et al.* [83], for example, showed that a droplet of highly rewarding sucrose induced broad changes in bumble bees’ behaviour, including more ‘optimistic’ responses to ambiguous stimuli and faster recovery from simulated predator attacks. Sucrose reward might have triggered a positive emotional state reducing the impact of a negative experience (see [84] for an alternative view). Furthermore, play behaviour has been proposed as a marker of positive emotions [44,85]. Bumble bees, for instance, voluntarily rolled wooden balls without apparent benefit such as food or mating [86]. The rewarding nature of this activity was further supported by bees’ ability to form a positive association between a neutral cue and ball rolling, suggesting hedonic value. Bumble bees, like vertebrates, may engage in inherently pleasurable activities, indicating that positive states can arise without external rewards [82]. Fruit flies also appear to engage in play-like behaviour, reminiscent of children enjoying swings on a playground [87,88]—some flies voluntarily and repeatedly climbed onto a rotating platform despite no reward, fulfilling key criteria of play. Studies in flies now allow for detailed investigation of the genetic, neuronal and biochemical mechanisms underlying such playful behaviour and its potential benefits [88].

**Box 1. Emotion-like states in fruit flies: integrating behavioural and neurobiological evidence**

Research in fruit flies (*D. melanogaster*) illustrates how persistent, valenced emotion-like states can be inferred by combining behavioural observations with neurobiological mechanisms. Two phenomena, learned helplessness and a depression-like state, highlight the value of this integrative approach.

— **Learned helplessness**

In this paradigm, flies are exposed to aversive stimulation (heat or electric shocks) which is either dependent on their behaviour (‘in-control’) or independent of it (‘yoked’). Flies subjected to uncontrollable stress progressively reduce their locomotor activity, pause more frequently and eventually stop making escape attempts. These effects persist beyond the period of stress exposure and are interpreted as indicative of learned helplessness [77,89].

— **Depression-like state**

Prolonged vibration stress selectively reduces voluntary (spontaneous walking) and reward-seeking behaviours (courtship initiation, response to sweet stimuli), while reactive behaviours such as phototaxis remain intact [90]. This dissociation suggests a depression-like state characterized by reduced motivation and anhedonia rather than global motor impairment.

— **Neurobiological mechanisms and rescue**

Reduced motivation and anhedonia are accompanied by decreased levels of serotonin and dopamine in the brain. Importantly, these changes are reversible—antidepressant treatments (e.g. fluoxetine, lithium and 5-HTP) restore both behaviour and neurochemical balance [90]. Circuit-level analyses identify reduced serotonergic signalling in the mushroom bodies as a key mechanism [90]. Hermanns *et al.* [89] further identified a neuromodulatory cascade by which sucrose produces antidepressant-like effects; octopaminergic neurons in the suboesophageal zone activate dopaminergic PAM neurons, which recruit serotonergic DPM neurons projecting to the mushroom bodies. This pathway restores serotonin release and rescues behaviour, linking metabolic state, reward and affective regulation.

Together, these findings show how persistent and valenced behavioural changes supported by identifiable neural mechanisms can be used to infer emotion-like states in insects, illustrating the strength of integrating behavioural and neurobiological approaches.

Vocalizations are major channels to communicate emotions [91,92]. In humans, prosodic cues such as crying or laughter convey clear emotional valence and arousal [93], and many non-human species produce specific vocalizations reliably linked to emotional states [94,95]. For example, rats emit ultrasonic calls tied to positive or negative contexts [96,97]. Similar patterns occur in farm animals and primates [98,99]. The source–filter theory of vocal production helps explain this link [94,100,101]—the structure of vocalizations in mammals and birds depends on respiratory and phonatory anatomy, which emotional states can transiently affect through somatic and autonomic pathways, altering acoustic parameters associated with emotional intensity and/or arousal. Invertebrates, especially insects, also generate rich repertoires of acoustic and vibrational signals for defence, mating or predator avoidance [102]. Although mechanistically distinct, these signals may encode internal states, supporting Darwin's early view that emotional expression can emerge even through wing stridulation [103]. Honey bees, for example, respond to hornet attacks by retreating to the nest, reducing recruitment dances and emitting vibratory stop signals (figure 1) [60]. These signals prevent reductions in dopamine levels in the brains of both signallers and receivers, reducing both the perceived hedonic value of food and foraging activity; this effect is consistent with the role of dopamine as a ‘wanting’ system [104]. Vibro-acoustic signals, thus, represent promising indicators of emotion-like states in invertebrates [105], with potential applications for studying processes such as fear learning [106].

Facial expressions are key indicators of emotions. Ekman's pioneering cross-cultural work identified at least six universal basic emotions [107], and the development of the Facial Action Coding System (FACs) [108] provided an anatomically based framework for classifying emotional expressions. Analogous systems have since been applied to primates [109–111], dogs [112] and horses [113]. In invertebrates, however, a comparable measure of facial expression is not possible owing to the lack of facial muscles. Cephalopods, such as octopuses, cuttlefish and squids, might nonetheless signal internal states through rapid skin colour changes and dynamic epidermal structures [114]. Arthropods, constrained by their rigid facial cuticle, have limited expressive capabilities. Nevertheless, advances in high-resolution video and motion analysis now enable detection of subtle movements, such as those of the ligula and antennae, which may be potentially linked to emotional contexts. Berridge's work on ‘liking’ responses in rats showed that fine orofacial reactions to sucrose, such as rhythmic tongue protrusions, lateral tongue movements, lip smacking and paw licking, reliably reflect hedonic value rather than mere sensory response [115,116]. Could analogous fine-scale movements in bees, such as proboscis extension, specific tongue dynamics or subtle antennal and mouthpart motions in response to sucrose or quinine, similarly provide a window into affective valence? Supporting this idea, odours previously paired with sucrose elicit forward, antennal movements, whereas alarm pheromones trigger backward, diverging ones [117]. While not direct evidence of emotion, these fine-scale motor patterns may serve as sensitive behavioural markers of stimulus valence.

## 4. Cognitive approach

The cognitive approach to emotions is particularly relevant as it links external events to personal needs, values, goals and beliefs, offering deeper insights than behavioural evidence alone [31,118]. Emotions emerge from appraisal processes rather than mere behavioural or physiological reactions. Through appraisals, experiences gain meaning in uncertain environments, guiding decisions and actions. The link between cognition and emotion is bidirectional—cognitive processes can trigger emotions, while emotional states, whether transient or persistent (mood), influence judgement, attention and memory. Such interactions likely occur in non-human animals as well, though they are harder to assess since human research often relies on language-based tools like self-reports, word-recognition tasks and scenario-based tasks. Nonetheless, cognitive biases such as judgement bias illustrate how paradigms from human research can be adapted to explore analogous processes in animals [119,120].

Originally developed for humans, the judgement bias task, an approach that assesses changes in decision-making under ambiguity that reflect the valence of internal states, has since been adapted, refined [121] and validated [119,120] for various non-human animals. To date, it has been applied to only three invertebrates from two insect orders: honey bees and bumble bees (Hymenoptera; [83,122–124]), and fruit flies (Diptera; [125]). Bateson *et al.* [122] and Schlüns *et al.* [123] examined negative judgement bias in honey bees using a Go/No-Go olfactory conditioning protocol, exploiting their natural proboscis extension reflex. During training, bees learned to associate one odour mixture (1 : 9 ratio of 1-hexanol to 2-octanone) with sucrose and another (9 : 1 ratio) with bitter quinine. After conditioning, half of the bees were shaken for 60 s to simulate a predator attack and then tested with intermediate odour mixtures (3 : 7, 1 : 1 and 7 : 3). Shaken bees were less likely to extend their proboscis to ambiguous odours resembling the quinine-associated ones, suggesting that shaking induces a negative cognitive bias toward uncertain cues. However, alternative explanations, such as enhanced discrimination driven by arousal and neuromodulators like octopamine, remain possible [42,126].

Solvi *et al.* [83] extended this approach to investigate positive emotion-like states in bumble bees using a free-flying Go/No-Go task. Bees were trained to locate a cylinder placed under a green card on the left or a blue card on the right, with one colour-location combination offering a 30% sucrose reward and the other offering only water. After mastering the discrimination, bees were tested with intermediate colour-position cues. To induce a positive state, half of the bees received an unexpected droplet of concentrated sucrose before entering the test arena. These bees approached ambiguous stimuli faster than controls, suggesting a positive judgement bias (figure 1). Critics, however, have suggested that such ‘optimistic’ responses might instead reflect a transient increased exploratory or foraging motivation after receiving a reward rather than an emotional shift [84].

Strang & Muth [127] offered a cognitive reinterpretation of these findings grounded in learning theory. They proposed that bees’ responses to ambiguous cues may result from changes in stimulus generalization gradients (the tendency for responses learned to one stimulus to extend to similar stimuli, decreasing with perceptual distance) rather than an altered perception of the uncertain. Specifically, they suggested that an unexpected reward reduces the peak shift effect [128], where the response peak shifts away from the unrewarded stimulus (S–). In their study, bees rewarded with sucrose before testing responded more to ambiguous cues but less to novel ones. When training minimized peak shift, either through absolute conditioning or by narrowing the perceptual distance between S+ and S–, differences between experimental and control bees disappeared. These results suggest that post-reward behaviour may reflect a narrowing of the inhibitory gradient around S–, increasing approach to ambiguous stimuli. Overall, these findings challenge emotion-based interpretations and highlight the need to control for generalization effects when using cognitive bias paradigms to infer affective internal states in animals.

To overcome some of the limitations of the Go/No-Go task, an alternative active choice (Go/Go) paradigm requires animals to choose between two response options, ensuring that each trial reflects an active decision process [119,129]. To date, only one study has applied this method in bees [124]. In this study, bumble bees were trained to associate two colours (blue and green) with high or low rewards in separate reward chambers and were then tested with three ambiguous colours intermediate between the two trained colours. Bees exposed to brief shaking or temporary confinement, simulating predation, were significantly less likely than controls to choose the chamber previously associated with the high reward, indicating a negative judgement bias. Signal detection theory and drift diffusion modelling supported this result, showing that stressed bees had a lower subjective estimate of high-reward probability. Despite increasing attention to invertebrate welfare, studies investigating the interplay between cognition and emotion remain remarkably scarce [22,130]. Within Hymenoptera, research is limited to honey bees and bumble bees, and in Diptera, only a single study has tested fruit flies. Deakin *et al.* [125] used an active choice olfactory task in a T-maze, where flies chose between two odours—one associated with a sugar reward (3-octanol) and another with a mild electric shock (4-methylcyclohexanol)—or an ambiguous 1 : 1 mixture. Flies subjected to brief mechanical shaking were significantly less likely to approach the ambiguous cue, consistent with a pessimistic judgement bias. However, shaken flies also showed a non-significant trend towards reduced approach to the positively conditioned odour, suggesting that stress may lower reward valuation and increase aversion to uncertainty. However, perceptual asymmetries could have influenced results, as the ambiguous odour mixture may have resembled the positive cue more closely than an intermediate one. Another limitation in fruit fly studies is the reliance on group-level testing. Since emotions are inherently private experiences, behavioural responses measured in groups may be influenced by social interactions. Developing individual-level protocols is therefore crucial to validate these findings. Given the powerful genetic tools and well-characterized neurobiology of *Drosophila*, this species offers a unique opportunity to investigate the molecular and neural pathways underpinning emotion-like processes.

## 5. Mechanism of emotions: insights from physiology and neurobiology

In humans and non-human animals, emotions are traditionally assessed through indices of sympathetic and parasympathetic activation [19,20,28]. Common measures of arousal include heart rate, respiration, peripheral temperature, blood pressure, neuroendocrine activity, electroencephalography (EEG) and neuroimaging. For instance, during negative or stressful events [131], sympathetic activation stimulates the adrenal medulla to release adrenaline and noradrenaline, increasing heart rate, vasoconstriction and rapid energy mobilization. In parallel, the hypothalamic–pituitary–adrenal (HPA) axis drives the secretion of ACTH and glucocorticoids, sustaining energy availability and amplifying the effects of catecholamines. By contrast, comparable studies in invertebrates remain limited, though functional analogues of the HPA axis have been proposed. Even *et al.* [132] suggested that stress induces the release of brain biogenic amines, which enhance arousal, cognition and stimulus sensitivity. In insects, neurosecretory cells of the corpora cardiaca (CC) release metabolically active hormones into the haemolymph, including corazonin (Crz), adipokinetic hormone (AKH) and possibly diuretic hormone I (DH), mobilizing energy reserves from the midgut and fat body. Peripheral hormones such as allatostatins, tachykinin-related peptides and insulin-like peptides further modulate gut motility and CC activity [132]. Stress-related stimuli should, thus, elicit measurable changes in heart rate, respiration, peripheral temperature and neuroendocrine activity (figure 1). In insects, several non-invasive physiological readouts are already available or readily adaptable. For instance, infrared thermography provides contact-free measurements of body surface temperature and has been successfully applied in taxa such as ants and bees [133,134]. Although many insects are ectotherms, species capable of regional endothermy, such as honey bees [135], can actively modulate thoracic temperature, making thermal measures particularly informative in affective contexts (figure 1). In addition, respiration rate and CO₂ output, commonly quantified using respirometry [136], offer well-established metabolic proxies that could be fruitfully integrated into emotional physiology paradigms. Heartbeat proxies, while technically more challenging, can also be obtained using thermistor and electrophysiological recordings [137]. While primarily reflecting arousal rather than valence, these measures remain a crucial component of emotional experience. Yet, they are rarely applied in invertebrate research, largely owing to practical challenges like small body size, rigid exoskeletons and open circulatory systems, which complicate cardiovascular measurements. Still, integrating non-invasive physiological metrics, such as peripheral body temperature and respiration, into behavioural paradigms is feasible and enriches multi-component studies of emotions.

Insects have long served as powerful model systems for investigating the neural mechanisms underlying behaviour. Particular attention has been devoted to biogenic amines, given their prominent roles as neuromodulators across diverse behavioural contexts [138]. Among their many functions, a key role of biogenic amines is to mediate reinforcement signals in circuits underlying associative learning. In many insect species, distinct biogenic amines convey signals related to reward and punishment, thereby acting as instructive inputs to sensory-processing circuits. Through their tight functional connectivity with these reinforcement pathways, sensory representations acquire specific valence, enabling organisms to form adaptive associations between environmental cues and their positive or negative consequences. In *D. melanogaster*, specific subsets of dopaminergic neurons (DANs) convey the reinforcing properties of appetitive and aversive stimuli by targeting distinct compartments of the mushroom bodies [139,140]. In hymenopterans such as honey bees and in crickets, the situation appears different: appetitive reinforcement depends primarily on octopamine (OA) signalling [141,142], whereas dopaminergic signalling mediates aversive reinforcement [143,144]. In these insects, pharmacological or RNAi-based blockade of octopaminergic [145] or dopaminergic transmission [143] selectively impairs appetitive or aversive associative learning, respectively, across different conditioning paradigms. These differences between bees and crickets on the one hand, and fruit flies on the other, indicate that functional homologies in aminergic reinforcement systems should be treated with caution. Despite these species-specific variations, insects clearly possess distinct neural pathways that assign positive or negative valence to events in a learning context. While this does not, in itself, demonstrate emotional experience in insects, it shows that even small brains can differentiate appetitive from aversive events, providing a neural foundation for discussions on the prerequisite of emotional processing.

Beyond providing instructive information on reward and punishment in an associative learning context, biogenic amines also shape behavioural responses to threat or conflict, which in vertebrates are often tied to affective states. In humans, their importance is supported by pharmacological treatments targeting aminergic systems to alleviate depression, anxiety and emotional dysregulation [146,147]. In crustaceans, serotonergic (5-HT) signalling modulates social dominance and aggression [148,149], a pattern mirrored in fruit flies, where 5-HT levels influence dominance hierarchies. In honey bees, defensive responses, culminating in stinging, are modulated by serotonin and dopamine, which regulate both the likelihood and intensity of attacks [150]. The large number of dopaminergic neuronal clusters identified in the honey bee brain suggests functional specialization within this system [151]. While some dopaminergic neurons are involved in aversive reinforcement signalling (see above), others are likely to contribute to the modulation of defensive behaviour, among others. Serotonin also modulates anxiety-like behaviours in invertebrates. In crayfish, serotonin injection or exposure to stressors (e.g. electric shock) induces light avoidance, reminiscent of anxiety, whereas anxiolytic drugs (e.g. chlordiazepoxide) or serotonin antagonists abolish these effects [70]. Interestingly, the serotonergic involvement occurs when anxiety is triggered by social harassment, as chlordiazepoxide or methysergide restore normal behaviour [71]. In crabs, acute exposure of fluoxetine (Prozac®) dissolved in the seawater tank reduced time spent in dark zones, suggesting anxiolytic effects. In *Drosophila*, Mohammad *et al.* [152] showed that flies exhibit thigmotaxis behaviour associated with anxiety. Heat shock or reduced dSerT mRNA or d5-HT1B expression increased this wall-following behaviour, while benzodiazepine (diazepam) or elevating d5-HT1B or dSerT mRNA expression reduced it, parallelling rodent anxiety models.

Biogenic amines also modulate arousal and motivation. As discussed above, forager honey bees facing predators retreat to the nest, reduce recruitment dancing and produce stop signals that reduce dopamine levels even in nestmates not directly exposed to danger. Strikingly, feeding bees with dopamine reversed these effects and reduced predator-specific behaviours [60]. Similarly, administering the dopamine precursor, L-DOPA, elevated brain dopamine levels, decreased aversion to predators and rescued olfactory sensitivity and learning [61], highlighting the role of dopamine as a positive ‘wanting system’ underlying appetitive motivation. Altogether, these findings underscore the multi-faceted roles of biogenic amines in shaping insect behaviour and identify aminergic systems as key modulators of invertebrate responses to life-threatening contexts. Thus, biogenic amines appear to occupy a central position in the neural regulation of survival-related responses.

This point is especially evident in cognitive experiments assessing judgement bias in bees (as discussed in §4), which provide compelling evidence for valence-based modulation of behaviour in insects. Bees exposed to aversive events such as shaking showed reduced aminergic signalling—haemolymph collected after simulated predator attacks contained lower levels of dopamine, octopamine and serotonin [122]. Conversely, unexpected sucrose rewards increased dopamine levels [83]. Topical application of the dopamine-receptor antagonist fluphenazine abolished this sucrose-induced optimistic bias and its buffering effect on defensive responses to simulated predator attacks. These dopaminergic changes are consistent with the engagement of a dopamine-based ‘wanting’ system driving appetitive motivation, an established function of dopaminergic neurons described above [104]. Together, these findings indicate that insect neuromodulatory systems mediate systematic, valence-dependent behavioural shifts closely paralleling affective modulation in vertebrates.

While these studies cannot provide direct insight into the subjective experience, they show that biogenic amines, in concert with other neuromodulators, form flexible systems assigning valence to sensory events and guiding adaptive responses to reward, punishment and threat [153]. Their involvement does not imply emotional homology across taxa but suggests that aminergic signalling supports fundamental functions, perhaps originating in motor control that evolution later co-opted for emotional phenomena [22,154]. The lack of anatomical homology with vertebrate structures such as the amygdala, central to generating primary emotional states [65,155,156], does not preclude functional analogies in invertebrates. Insects’ mushroom bodies and crustaceans’ hemiellipsoid bodies, along with their aminergic circuits, may perform valence labelling through reinforcement learning and threat modulation, approximating an emotional dimension within Pancrustacea. The key challenge does not lie in seeking one-to-one anatomical parallels with vertebrates, but in identifying the neural mechanisms and brain areas that underpin emotional processing in insects.

## 6. Conclusion and future directions

Current evidence from multi-dimensional studies suggests that insects possess core components of emotions, including arousal modulation, valence coding, persistence, generalization, flexibility and global coordination. Building on the functional approach [24,30], we argue that studying emotion-like processes requires a multi-component perspective rather than focusing on any single domain. Behavioural data alone cannot distinguish reflexive responses from genuine affective states, while exclusive reliance on neurophysiology risks reductionism. In particular, the bidirectional interplay between cognition and emotion warrants deeper investigation and the development of novel experimental paradigms.

From an evolutionary perspective, the presence of core emotional elements in phylogenetically distant species is unsurprising. Emotions likely offered early organisms strong adaptive advantages by enabling flexible responses to opportunities and threats. Even single-celled eukaryotes, aneural and simple neural animals possess rudimentary arousal and valence systems [157], which became increasingly complex during the Cambrian explosion with expanding ecological niches and predator–prey interactions. Sociality may have further refined these systems, as behavioural and postural by-products of internal states were co-opted for communication and eventually transformed into functional emotional signals. In social vertebrates, particularly great apes and humans, these systems reached their highest complexity, giving rise to emotional contagion and empathy.

The strongest evidence for emotion-like processes in invertebrates comes from crustaceans and insects, such as bees and fruit flies. Yet the field remains unbalanced; behavioural studies dominate, while integrative, multi-component approaches are rare. This limitation continues to fuel debate over whether invertebrate responses reflect simple reflexes, context-dependent adjustments or genuine affective states, an issue with major implications for ethics, welfare and policy. Future research should broaden the taxonomic scope, with particular emphasis on social invertebrates, such as hymenopterans, whose complex behaviours and accessible neural circuits make them ideal models. Investigating the social modulation of emotion, including communication and collective effects, will illuminate both evolutionary continuities and divergences with vertebrate systems. Tracing the evolution of emotional building blocks through integrative approaches can ultimately clarify how emotion-like systems emerged and diversified across the animal kingdom.

## Data Availability

This article has no additional data.

## Theme

One contribution of 19 to a theme issue ‘Mechanisms, development, phylogeny and functions of emotional expressions’.
